# Simultaneous Determination of Thirteen Q-Markers in Raw and Processed *Tussilago farfara* L. by UPLC-QQQ-MS/MS Coupled with Chemometrics

**DOI:** 10.3390/molecules24030598

**Published:** 2019-02-08

**Authors:** Liu Yang, Hai Jiang, Ajiao Hou, Xinyue Guo, Wenjing Man, Meiling Yan, Xudong Xing, Bingyou Yang, Qiuhong Wang, Haixue Kuang

**Affiliations:** 1 Key Laboratory of Chinese Materia Medica, Heilongjiang University of Chinese Medicine, Ministry of Education, Harbin 150040, China; hxk_yl@163.com (L.Y.); jianghai_777@126.com (H.J.); Hou_Ajiao@163.com (A.H.); m17645028606@163.com (X.G.); mm1532326@163.com (W.M.); hxk_yan@163.com (M.Y.); mrxing_xudong@126.com (X.X.); ybywater@163.com (B.Y.); 2 School of Traditional Chinese Medicine, Guangdong Pharmaceutical University, Guangzhou 528458, China

**Keywords:** UPLC-QQQ-MS/MS, *Tussilagofarfara* L., Q-markers, processing, chemometrics, quality assessment

## Abstract

The purpose of this study was to establish a rapid, reliable, and sensitive ultra-performance liquid chromatography with triple-quadrupole tandem mass spectrometry coupled with chemometric method to measure and evaluate the differences between thirteen compounds in raw and processed *Tussilago farfara* L. from different sources. This assay method was validated, and the results indicated that the calibration curves for the thirteen compounds had good linearity (R^2^ > 0.9990). The limits of detection and limits of quantification of the thirteen compounds ranged from 0.0012 to 0.0095 μg/mL and from 0.0038 to 0.0316 μg/mL, respectively. The relative standard deviations (RSD) of the intra- and inter-day precisions and stability ranged from 1.06 to 2.00%, 0.26 to 1.99%, and 0.75 to 1.97%, respectively. The sample recovery rates of the thirteen compounds with different concentrations were 94.47–104.06%. The chemometric results, including principal component analysis, hierarchical clustering analysis, three-dimensional analysis, and box plot analysis, indicated that there are significance differences in raw and processed *Tussilago farfara* L. The results of this study confirm that the proposed method is the first reported method that has been successfully applied for simultaneous determination and discovery of the difference between thirteen compounds of raw and processed *Tussilago farfara* L. Thus, this method could be a helpful tool for the detection and confirmation of the quality of traditional Chinese medicines and provide a basis for future pharmacological studies.

## 1. Introduction

Traditional Chinese medicines (TCMs) have played an important role in maintaining human health and treating diseases because of their long, historical applications and reliable therapeutic efficacy in many countries. In contrast to Western chemical medicines, many TCMs have different processing methods, such as steaming, boiling, honey-frying, stir-frying, simmering, baking, etc. [1]. Processing is the most commonly used method in the preparation of TCMs. These processes can greatly improve the cleanliness of natural Chinese herbal medicines, while also reducing the toxicity of toxic compounds, adjusting the drug’s medicinal properties, and increasing therapeutic effects, making them more suitable for clinical applications [2]. The therapeutic effect of TCMs change after processing. One probable explanation for this result is that one or several compounds change during such processing procedures [3]. Therefore, we speculate that the changed compounds are the effective active ingredients. In a recent report, the quality markers of TCMs (Q-markers) were found to be the core factors for quality evaluation and quality control of TCMs [4]. It has been suggested that such Q-markers should be derived from Chinese herbal ingredients and related to their function and should be qualitatively identified and quantified [5]. Thus, we use the effective active ingredients as Q-markers to quantify the TCMs.

Farfarae Flos (FF), derived from the dry bud of *Tussilago farfara* L. of the Compositae family, is a famous herbal medicine used in China with the Chinese name “Kuandonghua” [6]. As a TCM, FF has been widely used in China for over 2000 years and is widespread in northern China, northwestern China, Jiangxi, Hubei, Hunan, etc. FF was first identified in the “*Shen Nong Materia Medica*”, are cord and summary of Chinese pharmacopoeia, and it has been used for the treatment of various ailments, including coughs, bronchitis, and asthmatic conditions. The main chemical compounds of FF are flavonoids, terpenoids, phenolic acids, alkaloids, polysaccharides, volatile oils, etc. [7]. Modern pharmacological studies have shown that FF has antitussive, expectorant and anti-inflammatory [8], anti-tumor [9], neuroprotection [10], immune regulation [11], anti-oxidant [12], etc. effects. Among them, phenolic acids and terpenoids have a good inhibitory effect on inflammatory factors [13,14]. FF also contains a large amount of phenolic acids in a much larger amount than that of terpenoids. Flavonoids displaya variety of pharmacological activities [15]. However, Chinese pharmacopoeia only uses tussilagone as an indicator of quality control, which lacks scientific support. TCMs have complex components and complex component interactions that work together. Therefore, researchers should comprehensively analyze the active ingredients in FF and perform scientific and reasonable quality control on FF. Honey-frying is the most commonly used method in the processing of FF. After processing, the content of the effective compounds changes, making it more effective for moistening the lungs and relieving coughs. A large portion of the literature has shown that the content of phenolic compounds is higher and the anti-inflammatory effect is stronger after honey-frying [16,17,18,19,20]. Such compounds have been proven to display a variety of biological activities and pharmacological effects. To date, there have been few reports on the simultaneous determination of the three kinds of chemical components in FF. Therefore, we used thirteen compounds (gallic acid, neochlorogenic acid, chlorogenic acid, caffeic acid, cryptochlorogenic acid, 3,4-dicaffeoylquinic acids, hyproside, rutin, 4,5-dicaffeoylquinic acids, kaempferol-3-*O*-rutinoside, quercetin, kaempferol and tussilagone) as Q-markers to quantify and evaluate raw and processed FF from nine provinces in China including Anhui, Gansu, Hebei, Henan, Hubei, Hunan, Jiangxi, Shanxi, and Sichuan.

In previous research, a number of chromatography–mass spectrometry (GC-MS), high-performance liquid chromatography tandem ultraviolet detector (HPLC-UV), and ultra-high liquid chromatography coupled with Q-extractive mass spectrometry (UHPLC-Q Extractive) methods have been developed for the quantitative determination of FF [21,22,23]. Nonetheless, such methods have limitations, including the need for longer chromatographic run times and the consumption of large amounts of organic solvents [24]. Meanwhile, the detected compounds are limited due to scanning only in positive or negative ion mode. In contrast, ultra-performance liquid chromatography coupled with triple-quadrupole tandem mass spectrometry (UPLC-QQQ-MS/MS) has exhibited a number of advantages, including good selectivity, wide application range, reduced run times, rapid analyses, improved resolutions, and lower mobile phase costs [25]. In addition, conducting the simultaneous scanning of positive and negative ion patterns, the UPLC-QQQ-MS/MS method has been applied to determine seven phenolic acids, five flavonoids, and one terpenoid in raw and processed FF. To the best of our knowledge, UPLC-QQQ-MS/MS has not yet been employed for the analysis of FF.

The data were further analyzed using principal component analysis (PCA), hierarchical clustering analysis (HCA), three-dimensional analysis and box plot analysis to provide more information regarding the differences between raw and processed FF, as well as to evaluate the quality of FF. The results show that there are considerable differences between raw and processed FF. The content of the thirteen Q-markers increased after processing except for that of chlorogenic acid. All the thirteen Q-markers were successfully screened, identified, and quantified, and these methods also provide a new way to quickly and intuitionally analyze the differences between raw and processed TCMs.

Here, for the first time, an UPLC-QQQ-MS/MS method was developed and validated for the simultaneous quantitation of thirteen Q-markers in raw and processed FF obtained from different sources. Conducting the simultaneous scanning of positive and negative ion patterns, the developed method was applied to comprehensively determine seven phenolic acids, five flavonoids, and one terpenoid in FF. The chemometric results directly indicate that there are significant differences between raw and processed FF. In addition, the proposed method could be a helpful tool for detecting the quality of and revealing the difference between raw and processed TCMs.

## 2. Results

### 2.1. Optimization of Extraction Conditions

**Figure 1:**
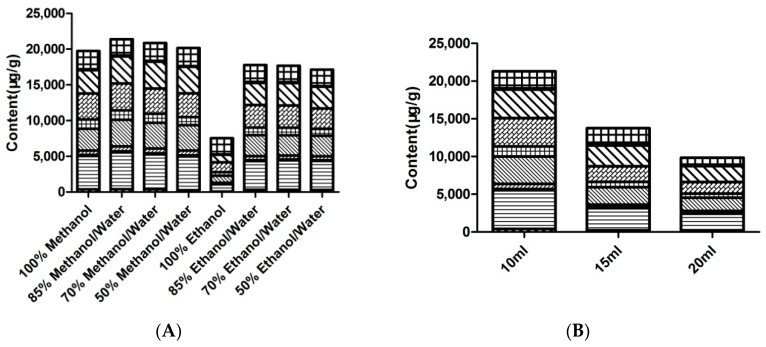

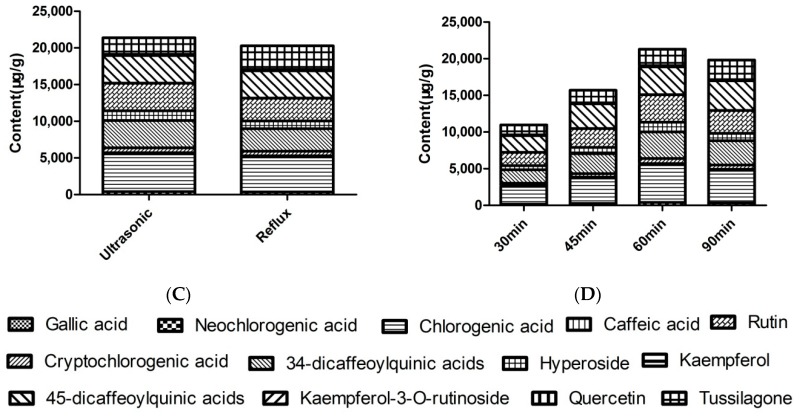
(**A**): Extraction efficiency of different solvents combinations; (**B**): Extraction efficiency of different solvent volumes; (**C**): Extraction efficiency of different extraction methods; (**D**): Extraction efficiency of different extraction times.

In order to ensure that the13 Q-markers in FF demonstrate high extraction efficiency, the key factors, including the extraction solvent, material ratio, extraction method, and extraction time, were optimized. First, a total of 0.05 g FF was added to 10 mL of different proportions of methanol (100% methanol, 85% methanol/water, 70% methanol/water, 50% methanol/water) and ethanol (100% ethanol, 85% ethanol/water, 70% ethanol/water, 50% ethanol/water) solutions and then subjected ultrasonic extraction for 60 min at room temperature. The results show that the extraction efficiency of 85% methanol/water is better than that of the other solvents. (Figure 1A, specific data were shown in Table S1A). Second, we optimized the material ratio, and a total of 0.05 g FF was added into 10 mL, 15 mL, and 20 mL of 85% methanol/water and subjected to ultrasonic extraction for 60 min at room temperature. The extraction efficiency decreased with the increase of the solvent (Figure 1B, specific data were shown in Table S1B). Therefore, 10 mL of 85% methanol/water was selected to be used as the solvent in this study. Third, ultrasonic extraction and reflux extraction were selected to optimize the extraction efficiency. Ultimately, the ultrasonic extraction was determined to be more effective than the reflux extraction (Figure 1C, specific data were shown in Table S1C). Thus, ultrasonic extraction was chosen to be performed in this experiment. Finally, the efficiency of different ultrasonic times (30 min, 40 min, 60 min, and 90 min) were investigated, and it was found that the extraction efficiency increases with the extraction time. However, when the extraction time exceeds 60 min, the extraction efficiency decreases (Figure 1D, specific data were shown in Table S1D). Thus, the optimal sample preparation method was as follows: A total of 0.05 g of FF was added into 10 mL of 85% methanol/water and subjected to ultrasonic extraction for 60 min (see Supplementary Materials Table S1 for specific data).

### 2.2. Optimization of Chromatographic and Mass Spectrometric Conditions

In preliminary experiments, chromatographic conditions such as column, mobile phase, solvent modifier, and gradient program were optimized in order to achieve optimal separation and the best peak shape in a short time. In this study, we optimized the Waters Acquity UHPLC HSS T3 column (50 mm × 2.1 mm, 1.8 μm) and Thermo Hypersil GOLD column (100 mm × 2.1 mm, 1.9 μm). When the Waters Acquity UHPLC HSS T3 column (50 mm × 2.1 mm, 1.8 μm) was used, the kaempferol and quercetin peaks were twisted, and the tailing was severe. Furthermore, the Thermo Hypersil GOLD column (100 mm × 2.1 mm, 1.9 μm) displayed peak symmetry and achieved rapid and complete separation for the thirteen Q-markers, especially for the same *m*/*z* isomer of phenolic acids. Methanol/water and acetonitrile/water were tested as the mobile phase, and the results show that the methanol/water solvent system has a higher response value. In addition, adding formic acid to the mobile phase not only ionizes certain compounds, but also improves the peak shape and reduces the tailing. Thus, we added 0.3% formic acid as a modifier in the mobile phase. The final optimized chromatographic conditions were performed on a Thermo Hypersil GOLD column (100 mm × 2.1 mm, 1.9 μm) with gradient elution for 25 min, and the mobile phase consisted of methanol (solvent A) and 0.3% (*v*/*v*) formic acid aqueous (solvent B) at a flow rate of 0.3 mL/min. The mass response of the thirteen Q-markers was studied in positive and negative mode, and the results are shown in Table 1. The mass spectrometry parameters were optimized in order to obtain higher signals for precursor and product ions. The precursor and product ions are shown in Table 1.

### 2.3. Method Validation

**Figure 2:**
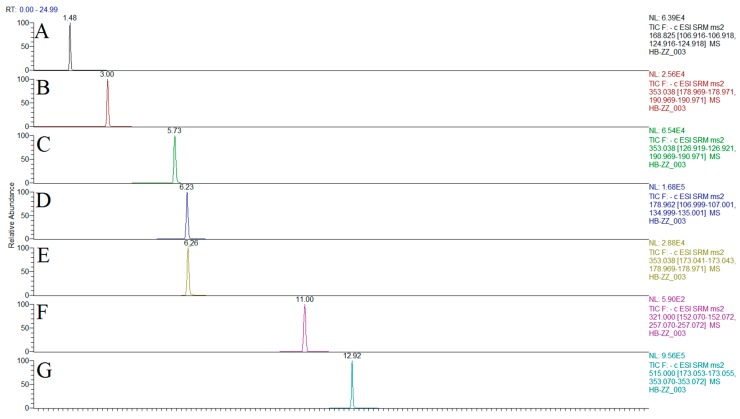

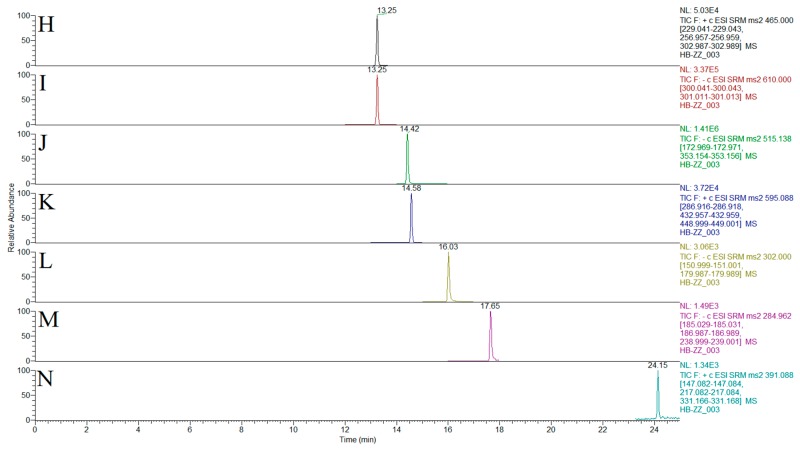
Chromatogram of the 13 the quality markers of traditional Chinese medicines (TCMs; all together: Q-markers) and internal standard (sequence numbers **A**–**N** are GA, 5-CQA, 3-CQA, CA, 4-CQA, chloramphenicol, 3,4-diCQA, hyperoside, rutin, 4,5-diCQA, kaempferol-3-*O*-rutinoside, quercetin, kaempferol, and tussilagone, respectively).

Under the above conditions of chromatography and mass spectrometry, the peaks of each component and internal standard appeared to be acceptable without interferences, indicating that the method had high selectivity. Representative UHPLC-QQQ-MS/MS chromatograms of the reference compounds and the samples are shown in Figure 2. Thus, this assay method was validated, and the results are displayed in Table 2 and Table 3. The calibration curves of the thirteen Q-markers had excellent linearity, and the correlation coefficients (R²) were higher than 0.9990. The limits of detection (LODs) and limits of quantification (LOQs) ranged from 0.0012 to 0.0095 μg/mL and from 0.0038 to 0.0316 μg/mL, respectively. The relative standard deviation (RSD, %) values of the intra- and inter-day precisions ranged from 1.06 to 2.00% and from 0.26% to 1.99%, respectively. The samples had good stability at 0, 2, 4, 8, 12, 24, and 48 h, and the RSD (%) ranged from 0.75 to 1.97%. The sample recovery rates and the RSD of the thirteen Q-markers with different concentrations were 94.47–104.06% and 0.16–4.43%, respectively. All the data showed that the developed UPLC-QQQ-MS/MS method was precise and accurate.

### 2.4. Quantitative Analysis

Each sample was analyzed in triplicate, and regression equations were used to calculate the contents of the thirteen Q-markers. The results are shown in Table 4. As can be seen in Table 4, the content of phenolic acids in FF is high, and the highest content is of chlorogenic acid. This was consistent with the results reported in the prior literature. Because the raw and processed FF are purchased from various Chinese herbal medicine markets, the processing method may be different, which is also the reason for the differences in the contents of the different batches. We plan to unify the processing methods in future experiments to reduce errors in the experiments. The content of tussilagone in FF in the 21 batches conformed to the pharmacopoeia standard, except for S9 and S11. This shows that the quality of FF on the market is unequal. The quality of FF from different batches should be further analyzed to eliminate accidental interference and optimize the quality of FF in the market.

### 2.5. Chemometrics Analysis

#### 2.5.1. Principal Component Analysis

**Figure 3:**
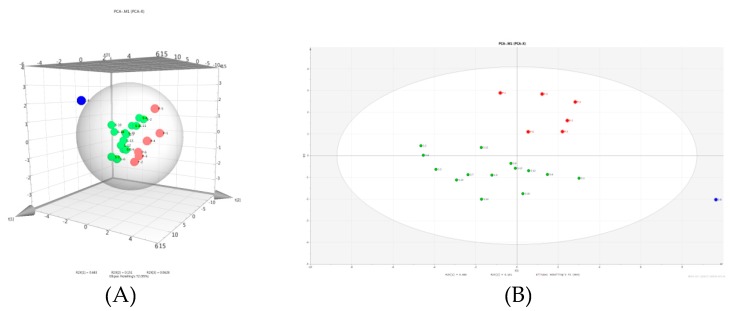
(**A**) Three-dimensional plot and (**B**) principal component analysis (PCA) plot of the 21 batches of raw and processed Farfarae Flos (FF).

Principal component analysis (PCA) is a multivariate mathematical statistical method that reduces dimensionality or converts multiple indicators into a few comprehensive indicators. It mainly eliminates the overlap and correlation in the chemical information through the dimensionality reduction of the data, and the multidimensional index becomes a simple index under the premise of losing little information, so that the data are simpler, more intuitive, and clearer [26]. PCA is widely used to evaluate the differences between the quality of Chinese herbal medicines and processed products [27]. The content of raw and processed FF was calculated in three dimensions. In Figure 3A,B, it can be seen that the 21 batches of samples were divided into two groups, with the green dots representing raw products and the red dots representing processed products. This directly reflects the significant differences between the raw and processed FF. S8 deviated from all the groups. There are several possible explanations for this result: one is that because we only studied 21 batches of FF samples and did not analyze all the origins of FF, the experimental results were accidental and uncertain. Another possible reason is that the FF samples we obtained may have been harvested in different seasons, which may have caused a significant difference in the content of the active components of the FF.

#### 2.5.2. Hierarchical Clustering Analysis

**Figure 4:**
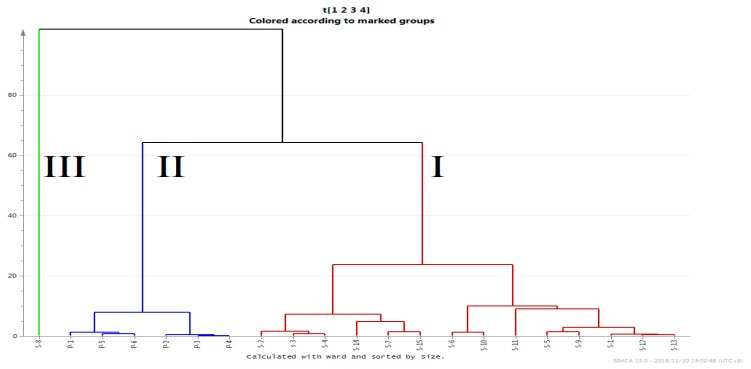
Dendrogram of the hierarchical cluster analysis (HCA) for the 21 batches of raw (S1–S15; group I) and processed (P1–P6; group II) FF. Group III included only S8.

Hierarchical cluster analysis (HCA) is a process for discovering useful information from a large amount of data. The basic principle is to distinguish different types of data according to the different characteristics of the data samples [28]. On the basis of the experimental data, the HCA method was used to comprehensively evaluate the 13 Q-markers of the raw and processed FF from different sources. First of all, we can see that the 21 batches were divided into three groups. Group I consisted of all raw products, with S2, S3, and S4 coming from Gansu Province, S5 and S9 from Hebei Province, S12 and S13 from Hubei Province, and the others from neighboring provinces with similar climates. Thus, they were all similar in content and were gathered into the same group. The processed products were gathered into group II, which was significantly different from the other groups. Because the S8 content was much higher than that of the other components, a separate group (group III) consisting only of S8 was formed (Figure 4). The results further demonstrate that the HCA method is a powerful tool for distinguishing raw and processed Chinese herbal medicines from different sources.

#### 2.5.3. Box Plot Analysis

**Figure 5:**
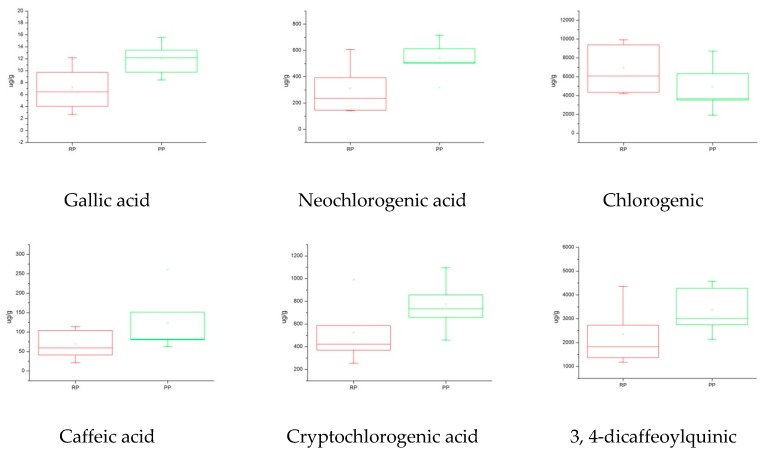

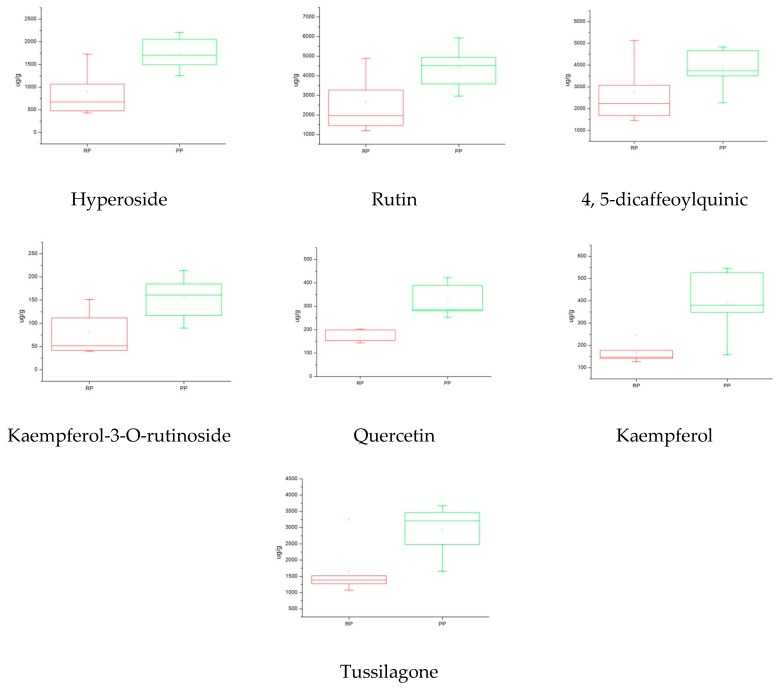
Comparative overview of the 13 Q-markers content in the raw (RP) and processed (PP) products of FF.

A box plot is a tool that can improve our understanding of quantitative information. It consists of five numerical points: minimum, lower quartile, median, upper quartile, and maximum. It can also add a mean to the box. There are always a variety of outliers in the real data, so in order not to cause the overall features to be offset, these outliers are drawn separately, and the two levels of the beard in the box plot are modified to the minimum and maximum observations [29]. In this experiment, the box plot was combined with PCA and HCA to analyze the quality of the raw and processed FF. It can be seen from the results of the box plot that the content of the thirteen Q-markers in the processed FF is significantly higher than that of the raw FF except with respect to chlorogenic acid (Figure 5). The results show that the established method is suitable for analyzing the differences between raw and processed FF from different sources by using 13 compounds as Q-markers. Moreover, it provides a new way to quickly and directly analyze the differences between raw and processed TCMs.

## 3. Materials and Methods

### 3.1. Chemicals, Reagents, and Materials

**Figure 6:**
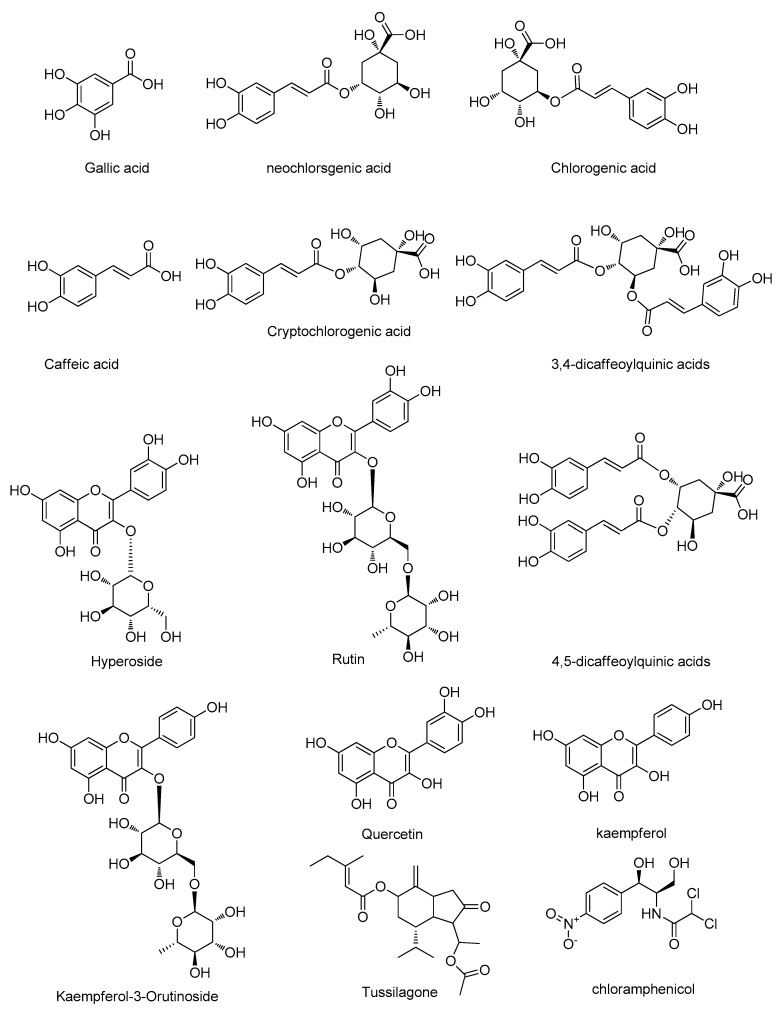
Structure of the 13 compounds and the internal standard, chloramphenicol.

HPLC-grade methanol was purchased from Fisher Scientific (Pittsburgh, PA, USA). Formic acid of HPLC-grade was purchased from Dikma Co. (USA), and the water was Wahaha purified water purchased from the Hangzhou Wahaha group (Hangzhou, China). Other reagents and chemicals were all of analytical grade. The reference standards of gallic acid (GA), neochlorogenic acid (5-CQA), chlorogenic acid (3-CQA), caffeic acid (CA), cryptochlorogenic acid (4-CQA), 3,4-dicaffeoylquinic acids, hyperoside (3,4-diCQA), rutin, 4,5-dicaffeoylquinic acids (4,5-diCQA), kaempferol-3-*O*-rutinoside, quercetin, kaempferol, and tussilagone and the internal standard (IS) of chloramphenicol were purchased from Chengdu Must Biotechnology (Chengdu, China). The structure of the thirteen Q-markers and IS are shown in Figure 6. All the reference standards and the IS had high purities, which were greater than 98%, and they were suitable for UPLC-QQQ-MS/MS analysis.

In the present study, 21 batches of raw and processed FF products were collected from the Chinese herbal medicine market in nine provinces (raw FF, S1–S15; processed FF, P1–P6 and the raw FF and the processed FF were matched. S1 matched P1, and S2 matched P2. S6 matched P6). These samples were identified by Professor Lianjie Su, and each of the 21 batches of FF voucher specimens were deposited at Heilongjiang University of Chinese Medicine, Harbin, China. All of the raw FF (S1–S15) and processed FF (P1–P6) were powdered and passed through an 80-mesh sieve. All the sample powders were deposited at a constant condition.

### 3.2. Chromatographic and Mass Spectrometric Conditions

Chromatographic analysis was performed in an ultra-high performance liquid chromatography system (Thermo Scientific TM, Vanquish TM, Waltham, MA, USA), consisting of an auto sampler and a binary pump. Chromatographic separation was achieved at 30 ℃ and performed on a Thermo Hypersil GOLD (Waltham, MA, USA) C18 column (100 mm × 2.1 mm, 1.9 µm). The mobile phase was composed of methanol (solvent A) and 0.3% (*v*/*v*) formic acid aqueous (solvent B) with a gradient elution for 0–5 min and 10–19% (A); 5–8 min and 19–25% (A); and 8–25 min and 25–89% (A). The flow rate of the mobile phase was kept at 0.3 mL/min, while the injection volume was 2 µL.

The UPLC system was carried out using a Thermo TSQ QUANTIS triple quadrupole mass spectrometer connected with an ESI interface. To gain more information on the structural identification, each sample was analyzed in both the positive and negative ion modes. The multiple reaction monitoring (SRM) conditions were optimized by infusion of the reference standard. The parameters in the source were set as follows: sheath gas of 35 Arb; aux gas of 8 Arb; ion transfer tube temperature of 325 °C; and vaporizer temperature of 350 °C.

### 3.3. Preparation of Sample Solutions

The dried powdered of the 21 batches FF samples (0.05 g) were weighed accurately, dissolved in 10 mL of 85% methanol/water solution using a 15 mL centrifuge tube, and extracted by ultrasonic extraction for 60 min at room temperature. The samples were subsequently centrifuged for 10 min at 5000 rpm. The supernatant was collected and filtered through a 0.22-μm filter membrane before analysis. All the solutions were stored at 4 °C until use.

### 3.4. Preparation of Standard Solutions

Stock solutions of the thirteen standard reference analytes were accurately weighed and dissolved in methanol solvent, achieving a final concentration of 1.000 mg/mL. The working standard solutions were then prepared by diluting the stock solution with 50% methanol/water solvent to a series of concentrations. The solutions were stored at 4 °C for further analysis.

### 3.5. Method Validation

The developed UPLC-QQQ-MS/MS method was validated by calibration curves, LODs and LOQs, precision, stability, and recovery [30]. A stock solution containing thirteen standard compounds was diluted to a series of appropriate concentrations with 50% methanol/water to establish calibration curves. Then, the calibration curve was constructed by the ratio of the peak area of the analyte to the IS to the concentration of the corresponding analyte solution. Each concentration was determined in triplicate. The limits of detection (LODs) and limits of quantification (LOQs) were determined by a signal-to-noise (S/N) ratio of 3 and 10, respectively. The analysis of the intra- and inter-day precisions of the method was evaluated with 6 replicate injections within one day (*n* = 6) and 3 consecutive days (*n* = 3), respectively. The intra- and inter-day precisions, expressed as relative standard deviations (RSDs), were less than 2.0%. The stability of the method was performed with 6repetitive injections at 0, 2, 4, 8, 12, 24, and 48 h under the same conditions, confirming the repeatability. Variations were expressed by RSD. In order to assess the accuracy of the method, three different concentration levels (50%, 100%, and 150%) of the thirteen standard solutions were added into 0.05 g of sample powder for recovery tests. According to the above method, the spiked samples were extracted and measured. Eventually, the average recovery was calculated by the formula: Recovery (%) = (amount found − un-spiked amount)/amount spiked × 100%, and RSD (%) = (SD/mean) × 100%.

### 3.6. Data Analysis

The data of the 21 batches of the tested samples were integrated with HCA, PCA, three-dimensional (SIMCA 13.0, Umetrics, Umeå, Sweden) analysis, and box plot analysis (OriginLab, Northampton, MA, USA). Each sample was analyzed in triplicate, and regression equations were used to calculate the contents of the thirteen Q-markers.

## 4. Conclusions

In this study, a rapid, reliable, and sensitive UPLC-QQQ-MS/MS method for simultaneous quantification and evaluation of the differences between thirteen Q-markers in raw and processed FF obtained from difference sources was developed for the first time. Satisfactory linearity, accuracy, precision, and stability in the concentration range were achieved. The chemometric results, including PCA, HCA, three-dimensional analysis, and box plot analysis, indicate that there are significant differences in the raw and processed FF. In summary, this study was sufficient to establish an UPLC-QQQ-MS/MS method combined with chemometrics analysis to simultaneous quantify and evaluate the differences between thirteen Q-markers in raw and processed FF, and this method could be a helpful tool for detecting the quality of traditional Chinese medicines and providing a basis for pharmacological studies. This method could also have a guiding effect with respect to clinical use of TCMs.

## Figures and Tables

**Table 1:** Optimized MRM parameters for the detection of the compound.

Compound	Structure	Polarity	Retention Time (min)	Precursor (*m*/*z*)	Product (*m*/*z*)	Collision Energy (V)
Gallic acid	C_7_H_6_O_5_	Negative	1.48	169	124	14
Neochlorogenic acid	C_16_H_18_O_9_	Negative	3.00	353	178	18
Chlorogenic acid	C_16_H_18_O_9_	Negative	5.73	353	191	16
Caffeic acid	C_9_H_8_O_4_	Negative	6.23	179	135	15
Cryptochlorogenic acid	C_16_H_18_O_9_	Negative	6.26	353	173	16
3,4-Dicaffeoylquinic acids	C_25_H_24_O_12_	Negative	12.92	515	353	18
Hyperoside	C_21_H_20_O_12_	Positive	13.25	465	302	13
Rutin	C_27_H_30_O_16_	Negative	13.25	609	300	36
4,5-Dicaffeoylquinic acids	C_25_H_24_O_12_	Negative	14.42	515	191	30
Kaempferol-3-*O*-rutinoside	C_27_H_30_O_15_	Positive	14.58	595	287	20
Quercetin	C_15_H_10_O_7_	Negative	16.03	301	151	21
Kaempferol	C_15_H_10_O_6_	Negative	17.65	285	186	29
Tussilagone	C_23_H_34_O_5_	Positive	24.15	391	331	10

**Table 2:** Calibration curves, linear range, limit of detection (LOD), limit of quantification (LOQ), precision, and repeatability for the thirteen Q-markers.

Compound	Calibration Curves	R^2^	Linear Range	LOD	LOQ	Precision	(RSD, %)	Stability
			(μg/mL)	(μg/mL)	(μg/mL)	Intra-Day (*n* = 6)	Inter-Day (*n* = 3)	(RSD, %)
Gallic acid	y = 16.698x + 0.1611	0.9996	0.0101–1.0080	0.0018	0.0060	1.98	1.47	1.89
Neochlorogenic acid	y = 31.38x + 0.108	0.9997	0.0105–8.3433	0.0020	0.0067	1.44	1.77	1.96
Chlorogenic acid	y = 15.725x − 0.0613	0.9995	0.0119–67.9039	0.0017	0.0056	1.24	1.64	1.71
Caffeic acid	y = 65.473x − 0.2765	0.9999	0.0111–6.6167	0.0017	0.0057	1.06	0.63	1.32
Cryptochlorogenic acid	Y = 39.925x + 0.2561	0.9991	0.0102–9.9099	0.0012	0.0038	2.00	0.56	1.86
3,4-Dicaffeoylquinic acids	y = 28.66x − 0.1627	0.9993	0.0230–38.6226	0.0044	0.0146	1.50	0.94	1.94
Hyperoside	y = 36.466x + 0.692	0.9994	0.0580–21.9959	0.0095	0.0316	1.19	1.82	1.90
Rutin	y = 14.183x − 0.0769	0.9994	0.0218–44.6006	0.0045	0.0149	1.66	1.79	1.59
4,5-Dicaffeoylquinic acids	y = 46.665x − 0.4	0.9992	0.0115–45.9080	0.0025	0.0083	1.81	1.99	1.95
Kaempferol-3-*O*-rutinoside	y = 46.092 − 0.1188	0.9999	0.0123–2.4550	0.0014	0.0049	1.94	0.26	1.97
Quercetin	y = 1.3638x + 0.1916	0.9996	0.0131–2.6147	0.0036	0.0120	1.10	1.15	1.63
Kaempferol	y = 2.2674x + 0.0698	0.9994	0.0194–0.9681	0.0030	0.0099	1.54	0.64	1.27
Tussilagone	y = 5.0377x + 0.2941	0.9994	0.0202–18.1436	0.0043	0.0145	1.96	1.05	0.75

**Table 3:** Recovery rates and the relative standard deviation (RSD) of the thirteen Q-markers with different concentrations.

Compound	Un-spiked (μg/mL)	Spiked (μg/mL)	Found (μg/mL)	Recovery (%)	RSD (%, *n* = 3)
Gallic acid	0.0494	0.0247 0.0494 0.0707	0.0745 0.0965 0.1205	101.89 95.45 100.65	2.04 4.43 2.37
Neochlorogenic acid	1.0509	0.5252 1.0504 1.4630	1.5896 2.0834 2.5246	102.57 98.29 100.73	1.57 1.89 1.07
Chlorogenic acid	15.4059	7.7050 15.4100 23.0000	22.6848 30.2700 38.3895	94.47 96.46 99.93	1.39 4.18 1.33
Caffeic acid	0.4516	0.2261 0.4522 0.6630	0.6767 0.9065 1.1043	99.53 100.59 98.44	0.92 1.01 0.41
Cryptochlorogenic acid	1.8639	0.9622 1.8644 2.8420	2.8472 3.7046 4.6697	102.20 98.73 98.73	2.23 0.93 1.55
3,4-Dicaffeoylquinic acids	12.0005	5.9993 11.9986 17.9350	17.8274 24.0563 29.7821	97.13 100.48 99.1448	1.26 1.45 2.82
Hyperoside	3.7520	1.8764 3.7528 5.5680	5.6739 7.5073 9.4219	102.42 100.07 101.83	1.01 2.04 1.30
Rutin	9.8986	4.9512 9.9024 14.8240	14.8921 19.6667 24.9019	100.85 98.64 101.21	1.82 2.20 1.47
4,5-Dicaffeoylquinic acids	12.5749	6.2909 12.5818 18.9200	18.6837 25.3422 31.3067	97.11 101.47 99.01	1.19 1.49 2.06
Kaempferol-3-*O*-rutinoside	0.2668	0.1334 0.2668 0.3690	0.4056 0.5330 0.6251	104.06 99.76 97.08	0.16 4.21 1.06
Quercetin	0.8109	0.4050 0.8099 1.1790	1.2234 1.5868 1.9852	101.84 95.79 99.60	1.59 2.45 1.43
Kaempferol	0.0202	0.0101 0.0202 0.0291	0.0304 0.0400 0.0487	100.85 98.10 98.60	1.54 2.84 1.86
Tussilagone	4.5558	2.2802 4.5604 6.8680	6.8484 8.9890 11.4616	100.55 97.21 100.55	0.53 1.61 0.59

**Table 4:** The contents of the thirteen Q-markers of raw and processed FF from different sources (μg/g, mean ± SD, *n* = 6).

NO.	Type	Source	Gallic Acid	Neochlorogenic Acid	Chlorogenic Acid	Caffeic Acid	Cryptochlorogenic Acid	3,4-Dicaffeoylquinic acids	Hyperoside	Rutin	4,5-Dicaffeoylquinic Acids	Kaempferol-3-*O*-rutinoside	Quercetin	Kaempferol	Tussilagone
S1	Raw	Anhui	8.18 ± 0.09	607.73 ± 2.03	9927.39± 47.67	103.59 ± 0.29	990.74 ± 3.78	4360.72 ± 12.36	1726.83 ± 7.82	4887.46 ± 28.20	5130.68 ± 20.70	151.41 ± 0.48	198.53 ± 0.99	16.24 ± 0.07	3243.53 ± 7.63
S2	Raw	Gansu	2.69 ± 0.07	234.59 ± 0.47	4331.77 ± 22.01	59.28 ± 0.12	370.29 ± 0.56	1828.17 ± 28.98	672.19 ± 1.2	1951.95 ± 19.89	2234.61 ± 23.73	51.21 ± 0.68	153.22 ± 2.76	17.75 ± 0.08	1381.59 ± 12.14
S3	Raw	Gansu	4.02 ± 0.02	393.21 ± 0.35	939040 ± 21.84	80.82 ± 0.50	586.17 ± 1.01	2734.77 ± 2.88	1052.15 ± 14.79	3097.38 ± 5.55	3070.06 ± 7.04	86.87 ± 1.12	173.01 ± 2.76	14.71 ± 0.02	1517.78 ± 14.60
S4	Raw	Gansu	12.17 ± 0.07	347.68 ± 1.58	7793.00 ± 29.02	113.75 ± 0.86	531.30 ± 2.49	2600.53 ± 16.70	1066.53 ± 10.59	3267.12 ± 24.47	3075.64 ± 22.59	111.75 ± 1.17	202.73 ± 0.51	24.51 ± 0.08	1489.64 ± 7.65
S5	Raw	Hebei	6.46 ± 0.02	14.6.03 ± 0.59	4203.92 ± 10.61	40.91 ± 0.34	421.93 ± 1.91	1370.87 ± 7.38	476.98 ± 3.49	1452.31 ± 11.23	1684.46 ± 9.58	39.24 ± 0.32	153.65 ± 0.85	12.65 ± 0.06	1268.35 ± 6.72
S6	Raw	Jiangxi	11.00 ± 0.04	141.88 ± 0.09	6068.68 ± 57.19	21.21 ± 0.25	254.16 ± 0.37	1178.55 ± 16.95	430.49 ± 0.19	1184.75 ± 16.58	1463.33 ± 4.88	41.32 ± 0.36	143.53 ± 1.20	14.17 ± 0.02	1075.73 ± 1.04
S7	Raw	Anhui	7.92 ± 0.01	585.79 ± 2.54	9902.63 ± 26.02	214.14 ± 0.96	1089.722 ± 4.08	4538.77 ± 32.61	1792.32 ± 8.27	5048.76 ± 27.07	5714.80 ± 32.95	156.65 ± 0.82	188.98 ± 0.55	118.06 ± 0.02	2813.91 ± 28.73
S8	Raw	Gansu	10.95 ± 0.08	958.48 ± 8.36	13468.97 ± 27.20	1152.00 ± 10.30	1936.99 ± 18.15	7556.77 ± 45.95	4344.47 ± 43.47	8996.29 ± 54.20	9259.64 ± 25.92	443.81 ± 6.09	393.01 ± 5.18	10.00 ± 0.02	3439.28 ± 13.47
S9	Raw	Hebei	7.38 ± 0.15	337.70 ± 1.34	5948.28 ± 18.53	74.36 ± 0.30	579.03 ± 2.48	2539.19 ± 6.82	839.86 ± 4.42	2369.20 ± 18.98	2869.91 ± 8.56	63.75 ± 0.24	146.36 ± 1.67	1204 ± 0.06	158.43 ± 9.12
S10	Raw	Henan	10.73 ± 0.01	728.21 ± 1.60	12312.28 ± 31.65	180.96 ± 1.81	1236.64 ± 4.92	5529.51 ± 20.33	2167.7 ± 8.13	6110.41 ± 8.52	5963.36 ± 22.59	206.87 ± 0.62	209.44 ± 0.10	37.89 ± 0.07	2377.36 ± 13.95
S11	Raw	Hubei	8.65 ± 0.08	927.03 ± 8.28	6292.18 ± 52.36	359.61 ± 3.77	1141.205 ± 11.69	4345.74 ± 38.25	1056.35 ± 5.68	2605.27 ± 15.17	5042.55 ± 15.07	84.86 ± 0.75	242.77 ± 0.99	28.10 ± 0.18	831.4229 ± 12.57
S12	Raw	Hunan	8.38 ± 0.06	543.96 ± 1.60	10008.91 ± 46.12	170.36 ± 0.73	851.02 ± 2.67	388560 ± 12.41	1652.48 ± 5.10	4188.77 ± 16.42	4542.47 ± 15.07	155.70 ± 0.92	204.17 ± 0.81	30.72 ± 0.09	2419.28 ± 12.64
S13	Raw	Hunan	9.54 ± 0.11	487.71 ± 4.11	8015.03 ± 48.09	134.59 ± 0.99	797.60 ± 6.38	3784.61 ± 28.35	1651.99 ± 4.16	4202.34 ± 21.58	4505.70 ± 15.86	140.97 ± 0.18	176.03 ± 1.53	26.38 ± 0.07	1791.41 ± 8.66
S14	Raw	Shanxi	3.83 ± 0.04	425.46 ± 4.72	8596.92 ± 76.78	358.54 ± 2.53	724.24 ± 3.67	3335.88 ± 20.81	1715.31 ± 8.05	1611.00 ± 2.85	3676.03 ± 23.38	36.51 ± 0.07	176.02 ± 1.77	11.53 ± 0.01	1121.69 ± 11.36
S15	Raw	Sichuan	5.84 ± 0.04	381.73 ± 4.64	8730.85 ± 61.76	247.74 ± 3.58	479.23 ± 3.98	2237.77 ± 15.14	706.65 ± 8.75	1536.99 ± 10.82	2400.77 ± 14.58	41.36 ± 0.46	166.45 ± 1.12	10.18 ± 0.01	1487.18 ± 9.86
P1	Processed	Anhui	8.47 ± 0.02	715.19 ± 2.55	8724.37 ± 26.62	62.94 ± 0.25	1096.91 ± 9.57	456.1.27 ± 47.51	2202.94 ± 17.89	5930.70 ± 24.53	4832.10 ± 49.77	213.80 ± 2.35	284.98 ± 2.35	39.32 ± 0.02	3461.47 ± 2.73
P2	Processed	Gansu	13.43 ± 0.11	319.78 ± 1.73	3664.34 ± 12.29	81.41 ± 0.89	457.58 ± 4.19	2135.04 ± 3.20	1252.22 ± 6.09	2956.19 ± 15.76	2273.09 ± 34.22	89.77 ± 0.66	252.50 ± 0.66	34.76 ± 0.09	2491.75 ± 7.10
P3	Processed	Gansu	12.72 ± 0.06	598.07 ± 3.88	5396.67 ± 10.40	150.98 ± 1.31	854.98 ± 6.13	4283.08 ± 4.08	2056.89 ± 15.24	4522.43 ± 4.05	4664.70 ± 40.76	160.54 ± 1.18	280.93 ± 1.18	38.01 ± 0.11	3208.26 ± 4.73
P4	Processed	Gansu	9.77 ± 0.03	612.47 ± 1.96	6329.39 ± 37.03	80.54 ± 1.77	849.83 ± 4.38	3512.93 ± 6.72	1753.09 ± 1.08	4936.30 ± 16.36	3825.99 ± 11.42	184.88 ± 3.31	422.04 ± 0.62	52.52 ± 0.04	3244.09 ± 14.47
P5	Processed	Hebei	15.53 ± 0.22	508.90 ± 4.85	1918.04 ± 13.18	260.27 ± 2.88	659.68 ± 7.37	3010.42 ± 3.49	1702.01 ± 16.02	4520.64 ± 12.05	3742.64 ± 10.51	167.64 ± 2.18	388.34 ± 0.07	54.58 ± 0.31	3668.36 ± 5.39
P6	Processed	Jiangxi	12.17 ± 0.23	501.96 ± 4.43	3527.02 ± 15.90	99.8 ± 1.56	734.74 ± 9.01	2746.89 ± 5.45	1492.10 ± 5.20	3585.47 ± 22.56	3504.55 ± 37.80	116.71 ± 1.25	305.00 ± 1.25	15.81 ± 0.01	1654.00 ± 7.29

## Supplementary Materials

The supplementary materials are available online.
