# Supplementary material for: Simultaneous Determination of Thirteen Q-Markers in Raw and Processed Tussilago farfara L. by UPLC-QQQ-MS/MS Coupled with Chemometrics
Source: Molecules. 2019 Feb 8;24(3):598. doi: 10.3390/molecules24030598 (PMC6385167; doi:10.3390/molecules24030598)
Supplement: Supplementary file 1 [file molecules-24-00598-s001.pdf]

Support 1 (A). Extraction efficiency of different solvents combinations (μg/g, n=3)

	Methanol/Water								Ethanol /Water							
	100%		85%		70%		50%		100%		85%		70%		50%	
	Mean	SD	Mean	SD	Mean	SD	Mean	SD	Mean	SD	Mean	SD	Mean	SD	Mean	SD
Gallic acid	14.46	0.59	20.27	0.94	18.21	0.42	11.87	0.14	2.10	0.11	17.6	0.20	8.19	0.44	1.28	0.89
Neochlorogenic acid	365.70	4.28	388.52	5.68	370.68	2.04	254.15	1.38	65.15	3.26	303.05	4.97	307.78	1.89	292.65	0.77
Chlorogenic acid	4673.11	39.49	5142.08	22.98	4885.84	31.50	4729.05	27.05	1020.50	1.80	4023.94	7.74	4133.99	6.04	4077.16	22.29
Caffeic acid	144.95	0.80	153.93	1.35	145.77	1.21	135.48	1.26	39.12	0.44	124.62	0.67	122.34	0.32	102.27	2.74
Cryptochlorogenic acid	603.43	49.35	678.81	28.46	667.69	4.91	664.46	4.19	169.05	0.75	556.18	5.50	550.29	1.02	528.57	22.32
3,4-dicaffeoylquinic acids	3066.75	5.01	3722.77	79.96	3610.57	15.17	3573.12	60.88	988.52	30.09	2937.94	11.52	2825.01	34.02	2863.09	11.38
Hyperoside	1288.50	10.66	1317.51	1.82	1239.51	5.97	1126.71	8.16	503.75	3.29	1075.92	1.08	1062.66	2.16	975.18	41.18
Rutin	3653.78	36.80	3769.77	6.49	3548.19	19.48	3336.50	24.80	1384.94	17.42	3161.55	0.91	3119.60	3.28	2858.19	13.13
4,5-dicaffeoylquinic acids	3246.64	23.71	3771.38	38.06	3746.37	41.80	3705.61	21.17	1049.45	3.99	3052.21	12.52	3119.29	5.81	3035.56	15.51
Kaempferol-3-O-rutinoside	102.55	4.96	108.08	0.25	94.28	0.41	92.40	0.44	34.72	0.49	78.77	1.43	81.64	1.20	87.12	0.52
Quercetin	38.46	0.96	61.33	0.21	42.06	1.63	-	-	32.22	1.75	64.50	0.69	56.74	1.90	-	-
Kampferol	36.26	0.07	30.45	0.28	24.04	0.38	13.27	0.24	17.73	0.22	27.06	0.18	24.64	0.03	20.83	0.91
Tussilagone	2543.31	3.32	2250.00	7.96	2483.04	31.02	2529.75	20.76	2229.55	16.60	2390.81	1..3	2289.14	11.30	2319.36	10.36

Support 1(B). Extraction efficiency of different solvent volume (μg/g, n=3)

	10ml		15ml		20ml	
	Mean	SD	Mean	SD	Mean	SD
Gallic acid	20.27	0.94	11.32	0.20	8.45	0.86
Neochlorogenic acid	388.52	5.68	206.89	1.02	157.23	1.14
Chlorogenic acid	5142.08	22.98	2934.90	12.02	2251.54	14.05
Caffeic acid	153.93	1.35	85.59	3.37	61.99	2.17
Cryptochlorogenic acid	678.81	28.46	380.26	2.86	292.67	2.20

**3,4-dicaffeoylquinic acids**	3722.77	79.96	2326.17	22.33	1767.51	14.88
**Hyperoside**	1317.51	1.82	716.74	5.64	524.58	7.33
**Rutin**	3769.77	6.49	2066.58	16.16	1561.44	14.57
**4,5-dicaffeoylquinic acids**	3771.38	38.06	2732.29	10.79	2104.85	18.39
**Kaempferol-3-O-rutinoside**	108.08	0.25	61.88	0.47	48.34	0.80
**Quercetin**	61.33	0.21	44.55	0.29	35.22	1.17
**Kampferol**	30.45	0.28	20.59	0.11	15.33	0.15
**Tussilagone**	2250.00	7.96	2193.46	30.72	1034.05	19.66

Support 1 (C). Extraction efficiency of different extraction method (μg/g, n=3)

	**Ultrasonic**		**Reflux**	
	**Mean**	**SD**	**Mean**	**SD**
**Gallic acid**	20.27	0.94	18.35	0.09
**Neochlorogenic acid**	388.52	5.68	354.66	1.86
**Chlorogenic acid**	5142.08	22.98	4755.95	41.15
**Caffeic acid**	153.93	1.35	146.59	0.89
**Cryptochlorogenic acid**	678.81	28.46	643.60	5.82
**3,4-dicaffeoylquinic acids**	3722.77	79.96	3074.35	4.29
**Hyperoside**	1317.51	1.82	1056.36	10.32
**Rutin**	3769.77	6.49	3110.76	23.25
**4,5-dicaffeoylquinic acids**	3771.38	38.06	3733.59	1.45
**Kaempferol-3-O-rutinoside**	108.08	0.25	73.83	1.16
**Quercetin**	61.33	0.21	96.96	0.22
**Kampferol**	30.45	0.28	30.33	0.00
**Tussilagone**	2250.00	7.96	3221.90	4.28

Support 1(D). Extraction efficiency of different extraction time (μg/g, n=3)

	30min		45min		60min		90min	
	Mean	SD	Mean	SD	Mean	SD	Mean	SD
Gallic acid	10.11	0.13	14.16	0.80	20.27	0.94	19.37	0.26
Neochlorogenic acid	175.27	1.06	251.39	1.22	388.52	5.68	318.13	2.78
Chlorogenic acid	2414.49	14.46	3478.53	16.26	5142.08	22.98	4440.22	55.88
Caffeic acid	75.29	0.49	102.04	0.49	153.93	1.35	136.25	2.32
Cryptochlorogenic acid	329.80	1.97	469.66	2.31	678.81	28.46	601.61	7.42
3,4-dicaffeoylquinic acids	1829.14	11.22	2732.03	12.94	3722.77	79.96	3302.94	22.73
Hyperoside	600.59	3.44	853.14	4.01	1317.51	1.82	1007.70	11.75
Rutin	1805.97	10.84	2592.60	12.53	3769.77	6.49	3122.30	33.05
4,5-dicaffeoylquinic acids	2267.12	14.31	3346.31	15.79	3771.38	38.06	4043.54	29.63
Kaempferol-3-O-rutinoside	43.75	0.32	62.46	0.29	108.08	0.25	70.86	5.56
Quercetin	54.92	0.31	57.43	1.15	61.33	0.21	65.96	0.37
Kampferol	16.97	0.08	25.67	0.10	30.45	0.28	28.98	0.67
Tussilagone	1354.92	8.22	1727.82	10.72	2250.00	7.96	2681.51	56.22
